# Efficient and assured reinforcement learning-based building HVAC control with heterogeneous expert-guided training

**DOI:** 10.1038/s41598-025-91326-z

**Published:** 2025-03-05

**Authors:** Shichao Xu, Yangyang Fu, Yixuan Wang, Zhuoran Yang, Chao Huang, Zheng O’Neill, Zhaoran Wang, Qi Zhu

**Affiliations:** 1 Northwestern University, Mccormick School of Engineering, Evanston, 60208 USA; 2 Department of Mechanical Engineering, Texas a&M University, College Station, 77843 Texas, USA; 3 Department of Operations Research and Financial Engineering, Yale University, New Haven, 06520 USA; 4 Department of Computer Science, University of Liverpool, Liverpool, L69 3BX UK

**Keywords:** HVAC control, Reinforcement learning, Deep learning, Electrical and electronic engineering, Mechanical engineering

## Abstract

Building heating, ventilation, and air conditioning (HVAC) systems account for nearly half of building energy consumption and $20\%$ of total energy consumption in the US. Their operation is also crucial for ensuring the physical and mental health of building occupants. Compared with traditional model-based HVAC control methods, the recent model-free deep reinforcement learning (DRL) based methods have shown good performance while do not require the development of detailed and costly physical models. However, these model-free DRL approaches often suffer from long training time to reach a good performance, which is a major obstacle for their practical deployment. In this work, we present a systematic approach to accelerate online reinforcement learning for HVAC control by taking full advantage of the *knowledge from domain experts in various forms*. Specifically, the algorithm stages include learning expert functions from existing abstract physical models and from historical data via offline reinforcement learning, integrating the expert functions with rule-based guidelines, conducting training guided by the integrated expert function and performing policy initialization from distilled expert function. Moreover, to ensure that the learned DRL-based HVAC controller can effectively keep room temperature within the comfortable range for occupants, we design a runtime shielding framework to reduce the temperature violation rate and incorporate the learned controller into it. Experimental results demonstrate up to 8.8*X* speedup in DRL training from our approach over previous methods, with low temperature violation rate.

## Introduction

Buildings account for around $40\%$of the energy consumption in the United States, of which nearly half is by the heating, ventilation, and air conditioning (HVAC) systems^1^. In addition, the operation of HVAC systems significantly affects the physical and mental health of building occupants, as people spend around $87\%$of their time indoors^2,3^, and even higher during the COVID-19 pandemic in recent years^4^. It is thus a critical task to develop effective HVAC control strategies that can maintain a comfortable indoor environment while reducing energy cost^5–7^.

In the literature, there are extensive works of developing *model-based approaches* for HVAC control. For instance, Maasoumy *et al.*^8^ use RC-networks to model the building thermal dynamics and applies the linear quadratic regulator (LQR) method for controlling the HVAC system. The work from Toub *et al.*designs a model predictive control (MPC) method to minimize the energy consumption and cost of the building HVAC system combined with a solar power unit. Some other works on model-based approaches can be found in papers^9–12^. However, to achieve good performance, these model-based approaches require the development of detailed and accurate physical models, which are often difficult and costly in practice. Thus, there has been significant interest in developing *learning-based, model-free approaches* for HVAC control, in particular those based on deep reinforcement learning (DRL). For example, the work from Wei *et al.*^13^utilizes the deep Q-learning method for controlling the indoor air flow rate and leverages the EnergyPlus platform^14^for simulation-based training. Various other techniques have also been applied for DRL-based building HVAC control, including Deep Deterministic Policy Gradient (DDPG)^15^, Proximal Policy Optimization (PPO)^16^, Asynchronous Advantage Actor-Critic (A3C)^17^, etc.

However, a major difficulty in adopting DRL-based methods to building HVAC control is that it could take a long time to train the RL agent in practice during building operation – note that in this case, the agent training is part of its live deployment. For instance, it may take more than 100 months of training to reach convergence for the Q-learning based methods^13,18^, and around 500 months of training for the DDPG algorithm to converge on a laboratory building model^19^. In Yu *et al.*’s work^20^, DDPG is used for temperature control and energy management, and it takes around $2.4 \times 10^4$ months to reach the best performance. Yu *et al.*^21^ present the method whose training time is almost $4 \times 10^4$ months in a multi-zone building environment. Clearly, such long training time would make it impossible to adopt DRL in practice for building control. While developing a detailed simulation model (e.g., in EnergyPlus) and conducting the training via simulation may help avoid this issue, the development of the simulation model itself is difficult and costly (in terms of both time and expertise), just as in the model-based methods.

Thus, recently researchers have been trying to improve the training efficiency for DRL-based building HVAC control. Xu *et al.*^3^ present a transfer learning approach to extract and transfer the building-agnostic knowledge from an existing DRL controller of a source building to a new DRL controller of a target building, and only re-train the building-specific components for the new DRL controller. The work from Lissa *et al.*^22^also leverages transfer learning, but for heat pump control in microgrid. However, the effectiveness of the transfer learning-based methods strongly relies on the similarity between the existing building target building and the transferred building, and may not be feasible when they are not similar^3^. There are also a few studies on the application of offline reinforcement learning for building HVAC control, where historical data on existing controllers are leveraged to train new RL-based controllers. For instance, the work from Schepers *et al.*^23^conducts conservative Q-learning (CQL) to train controllers for maintaining the room temperature setpoint. The problem of such offline RL methods, however, is that the learned agents’ performance strong depends on the quality of the historical data. And they tend to perform poorly due to the distributional shift between the historical data and the learned policy, and may have limited improvement even with fine tuning via online training^24^.

In this work, to address the above challenge in DRL training efficiency, we propose a **unified framework that leverages the knowledge from domain experts in various forms** to accelerate online RL for building HVAC control. This is motivated by the observation that in established domains such as building control, there is extensive domain expertise, represented in various forms such as 1) *abstract physical models*(e.g., RC-networks^25^or ARX models^26^) of building thermal dynamics – they are not accurate enough for enabling training DRL or designing model-based methods with good performance, but nevertheless contain valuable information of building dynamics, 2) *historical data* collected from existing controllers – they may not be able to train DRL controllers with good performance due to distribution shift, but also contain useful information on building behavior, and 3) expert rules that reflect basic policies. We believe that leveraging these domain expertise can help accelerate the online RL process. In particular, our framework first learns *expert functions* from existing abstract physical models and from historical data via offline RL, and then combines those with expert rules to generate an *integrated expert function*, which will then be used to drive online RL with prior-guided learning and policy initialization from expert function distillation. In experiments, our framework is able to significantly reduce the convergence time for DRL training by up to 8.8*X*, while maintaining similar performance (in terms temperature violation rate and energy cost).

Moreover, to further improve the learned DRL-based controller’s capability in keeping room temperature within the comfortable range, we propose a novel **runtime shielding framework** with an expert model. Instead of combining the temperature violation and the energy cost as the optimization objective (like during the DRL training), the framework considers the comfortable temperature range as constraints and tries to adjust the DRL-based controller’s output for meeting the temperature constraints during runtime. More specifically, the expert model takes the system state as input and predicts the next-step indoor temperature and worst-case indoor temperature in the next few steps. Based on such prediction, the framework iteratively adjusts the controller output for meeting the temperature constraints. The runtime framework provides a general design for reducing temperature violation rate, where various controllers can be incorporated. In this case, when our proposed DRL-based controller is incorporated into it, significant reduction in temperature violation rate can be observed in experiments.

To summarize, our work makes the following contributions:

- We propose a novel training framework to accelerate online RL for building HVAC control with heterogeneous expert guidances, including abstract physical models, historical data, and expert rules. These various guidances are unified in our framework via the expert functions.
- We propose a novel runtime shielding framework with an expert model that can further reduce the temperature violation rate when applying on our learned DRL-based controller.
- We conducted a series of experiments for evaluating the effectiveness of our framework. The results demonstrate that our approach can effectively reduce the DRL training time while maintaining low energy cost and temperature violation rate.

The rest of the paper is organized as follows. The second section discusses the related literature. The third section presents our approach, including the online DRL training framework with heterogeneous expert guidances and the runtime shielding framework. The fourth section presents the experimental results, and the last section concludes the paper.

## Related works

### Reinforcement learning for HVAC control

Building HVAC control is a critical and challenging problem as it significantly affects both building energy efficiency and occupants’ physical and mental health. In traditional model-based approaches, detailed and accurate physical models are needed for control optimization, but are often difficult and costly to develop and slow to run. Such limitations have motivated the exploration of model-free approaches in recent years, particularly those based on deep reinforcement learning^3,13,17,27^. These DRL-based HVAC control approaches leverage a variety of RL algorithms including DQN^13^, A3C^17^, DDPG^15^, PPO^16^, etc. For instance, Wei *et al.*^13^ convert the building HVAC control into a Markov decision process (MDP) problem and leverage the DQN method to intelligently learn the operation strategy based on offline simulations. Gao *et al.*^15^ adopt the neural network to predict occupants’ thermal comfort for part of their reward function design, and then apply the standard DDPG algorithm to learn from their building simulation environment. Abrazeh *et al.*^16^ develop a real-time digital twin with a PPO-based backstepping controller to maintain the relative humidity and temperature in buildings. However, a major obstacle in applying these DRL-based control algorithms is that they often require *dozens of months or more*for training to reach the desired performance^15,18,19^. Such long time is clearly not feasible for direct training during real building operation (i.e., sensing the real building environment and sending the actuation signals to HVAC equipment). It may be possible to avoid this by developing accurate and detailed building models and conducting training via simulations on tools such as EnergyPlus and Modelica^28^-based tools^18^, however, this again requires the development of those detailed and costly physical models and somewhat defeats the original purpose of using model-free approaches. Thus, it is critical to improve the efficiency of online RL for HVAC control *without* the development of detailed physical models.

### Transfer learning for HVAC control

One way to speed up RL is to transfer the learned policy between different buildings. For instance, the work from Xu *et al.*^3^ reduces the DRL training time by re-designing the learning objective and decomposing the neural network to a building-agnostic sub-network and a building-specific sub-network. The building-agnostic sub-network can be directly transferred from an existing DRL controller of a source building, and only the building-specific sub-network needs to be (re)-trained on the target building. This can reduce the DRL training time from months/years to weeks. Lissa *et al.*^22^ utilize the direct policy transfer between different houses with the same state/action space for heat pump control in microgrids. Zhang *et al.*^29^applies the transfer learning to a PPO-based controller for smart home to reduce the training cost. The main obstacles the current transfer learning-based methods face include the requirements for the source and target models to share the same learning objective, action space, and input space, and for the source and target buildings to be similar in their ambient conditions such as weather patterns. These requirements significantly limit the practical applicability of transfer learning-based methods, often resulting in poor performance when the target building is not similar to the source building or operates in a different environment^3^. In contrast, our approach presented in this paper provides an effective and efficient method to learn a policy with only expert knowledge on the building of interest. These two types of methods can be complementary in their usage scenarios.

### Offline reinforcement learning

Another way to accelerate online RL is through *offline RL*, by leveraging historical data collected under existing control policies. Recent offline RL works focus on two aspects: offline policy optimization, and offline policy evaluation. The former aims to learn an optimal policy for maximizing a notion of cumulative reward, while the latter is intended to evaluate the accumulated reward (or the value function) of a given policy. For offline policy optimization in particular, a major challenge is that the agent cannot directly explore the environment. And the error (called extrapolation error^30^) that is caused by selected actions not contained in the historical dataset could occur and propagate during the training. This is one of the reasons that limits the effectiveness of existing offline RL approaches for building HVAC control^23^. The approaches that address this challenge mainly utilize regularization or constraint-based methods to help the policy stay near to the existing actions in the historical dataset. For instance, the batch-constrained Q-learning (BCQ) approach^30^ restricts its action space to make the learned behavior similar to the actions in the historical dataset. Jaques *et al.*^31^ penalize divergence between the prior learned from the historical dataset and the Q-network policy using KL-control. The approach from Wang *et al.*^32^learns the policy by filtered behavioral cloning, which utilizes critic-regularized regression to filter out low-quality actions. And other related investigations can be found in papers^33–38^. From the prior experiments, we notice that not all offline RL algorithms can be chosen for building the expert function. The method like TD3+BC^38^ may not always provide a good value estimation for the given states, as it only aims to make the learned policy closer to the behavior in the offline dataset and tend to overestimate the Q-value. So in this work, we use historical data as one of the expert guidance and conduct offline RL to build an expert function. We leverage the idea from Kumar *et al.*^39^ to estimate the value function from historical dataset because of its effectiveness, by directly setting regularization on the Q-function and generating the Q-value estimation in a conservative way to reduce overestimation.

### Shielding methods for learning-based systems

Shielding methods typically first check a pre-defined shield and then adjust the control action accordingly by looking one step or a few steps ahead. For instance, to ensure safety, model predictive shielding^40,41^leverages a backup control policy to override the learning policy when unsafe scenarios are predicted to happen. In the Simplex architecture^42^, the high-assurance controller acts as a shield to the high-performance controller for improved system safety and performance. However, most shielding-based approaches rely on a fully-known environment model to synthesize a shield, which does not apply to our problem setting here where we focus on model-free building HVAC control. Moreover, shielding methods may also degrade the overall performance^43^. In this paper, we propose a novel runtime model-free shielding framework for the learned DRL-based controller. The framework does not require knowing the system dynamics, does not affect the training stage, is agnostic to the learned controller design, and as experiments show, can effectively reduce temperature violation rate while maintaining low energy cost.

## Our proposed approach

**Fig. 1:**
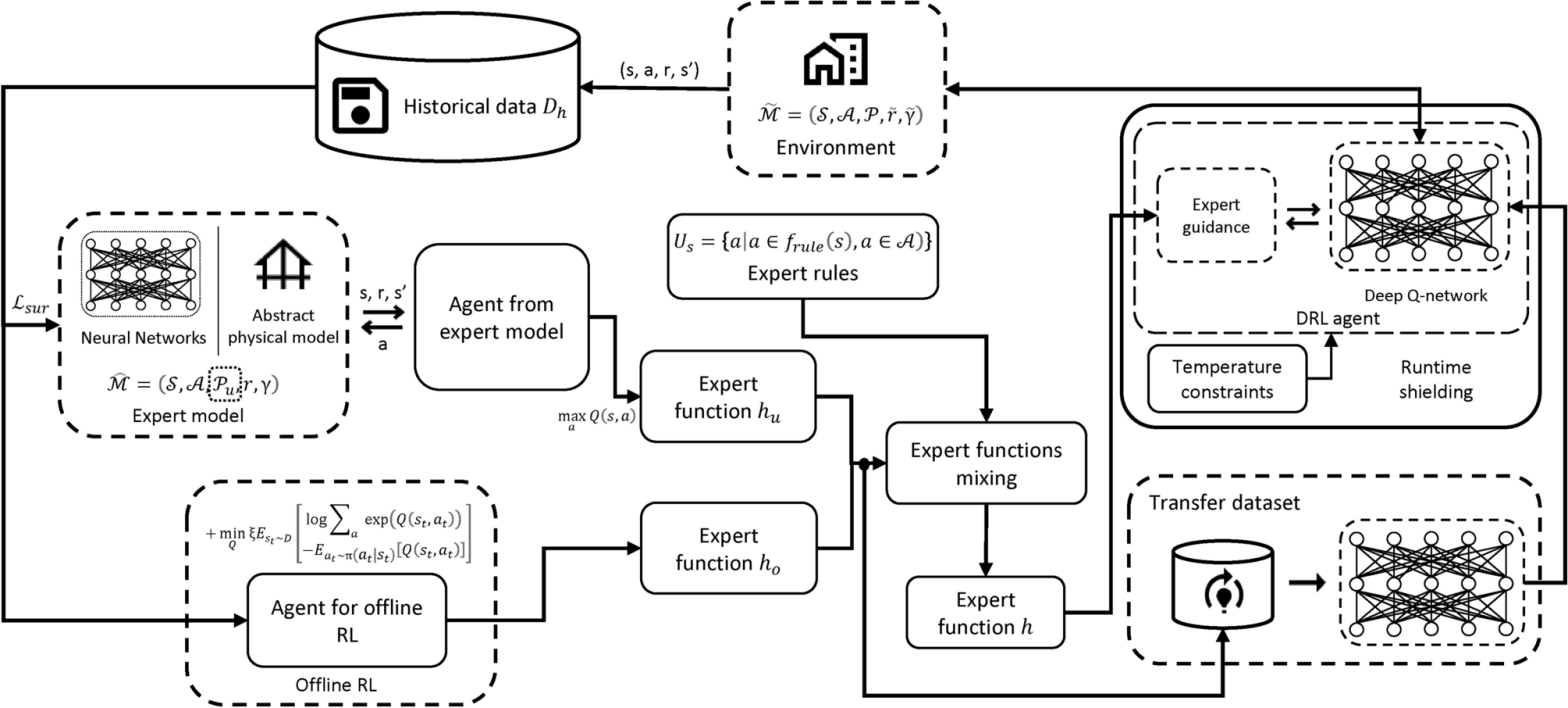
Overview of our online DRL training framework with heterogeneous expert guidances. The framework includes the following major components: (1) An expert function $h_u$ learned from an expert model, which can be an abstract physical model or a neural network with its parameters determined from a static historical dataset. (2) Another expert function $h_o$ learned from offline RL based on historical data. (3) An integrated expert function *h* generated by combining $h_u$ and $h_o$ as well as expert rules. (4) Application of prior-guided learning and policy initialization from expert function distillation based on *h*.

### System model

We use the building model with the fan-coil system from paper^18^, which is extended from a single-zone commercial building with manipulable internal thermal mass. The internal air is conditioned by an idealized fan coil unit (FCU) system, and the fan airflow rate is chosen from multiple discrete levels $\{f_1, f_2, \cdots , f_m\}$ (which can be viewed as *m* control actions; $f_1$ is to turn off the cooling system, and $f_m$ is to run it at full speed.). There are two different working modes in this system: the occupied time (daily from 7 am to 7 pm), and the unoccupied time (rest of the day). The HVAC system will run in a low-power mode during the unoccupied time for the energy-saving purpose (with the cooling system almost turned off). And the setting of comfortable temperature bound is different in these two modes. The system conducts control with a period of $\Delta t$. Each training episode contains two days of data, so there are $\frac{2880}{\Delta t}$ control steps in each episode. Other experiment-related settings can be found in experimental results section. The system state contains the following elements:

- Current physical time *t*,
- Indoor air temperature $T^{in}_{t}$,
- Outdoor air temperature $T^{env}_{t}$,
- Solar irradiance intensity $q^{sun}_{t}$,
- Power consumption during the current control interval $P_{t}$,
- Outdoor air temperature forecast in the next three control steps $\{T^{env}_{t+1}, T^{env}_{t+2}, T^{env}_{t+3}\}$, and
- Solar irradiance intensity forecast in the next three control steps $\{q^{sun}_{t+1}, q^{sun}_{t+2}, q^{sun}_{t+3}\}$.

One thing to note is that we add one additional variable in the implementation to the system state design, which is the remainder after dividing the current physical time *t* by $24*60*60$. This is to help the RL agent figure out the time position within one day (morning, noon, afternoon, etc.), and may help it reach better performance as observed in our preliminary experiments.

### Our online DRL training framework with heterogeneous expert guidances

As stated in introduction section, to accelerate online DRL for HVAC control, we propose a unified framework that leverages heterogeneous expert guidances including abstract physical models, historical data, and expert rules. Figure 1 shows the overview of our framework design. Specifically, the framework includes the following major components:

- An expert function $h_u$ learned from an expert model. The expert model could be an abstract physical model developed by domain experts (commonly exists in building domain), or in case such physical model is not available, a neural network with its parameters determined from historical data (but different from offline RL; more details later).
- Another expert function $h_o$ learned via offline RL on historical data that was collected using existing controllers.
- An integrated expert function *h* by combining $h_u$ and $h_o$ as well as expert rules.
- Application of prior-guided learning and policy initialization from expert function distillation based on *h*.

The detailed flow of our approach is in Algorithm 1. Next, we will first explain the underlying DRL algorithm we use, and then introduce the details of each component in our approach to improve DRL efficiency with heterogeneous expert guidance.

**Algorithm 1:**
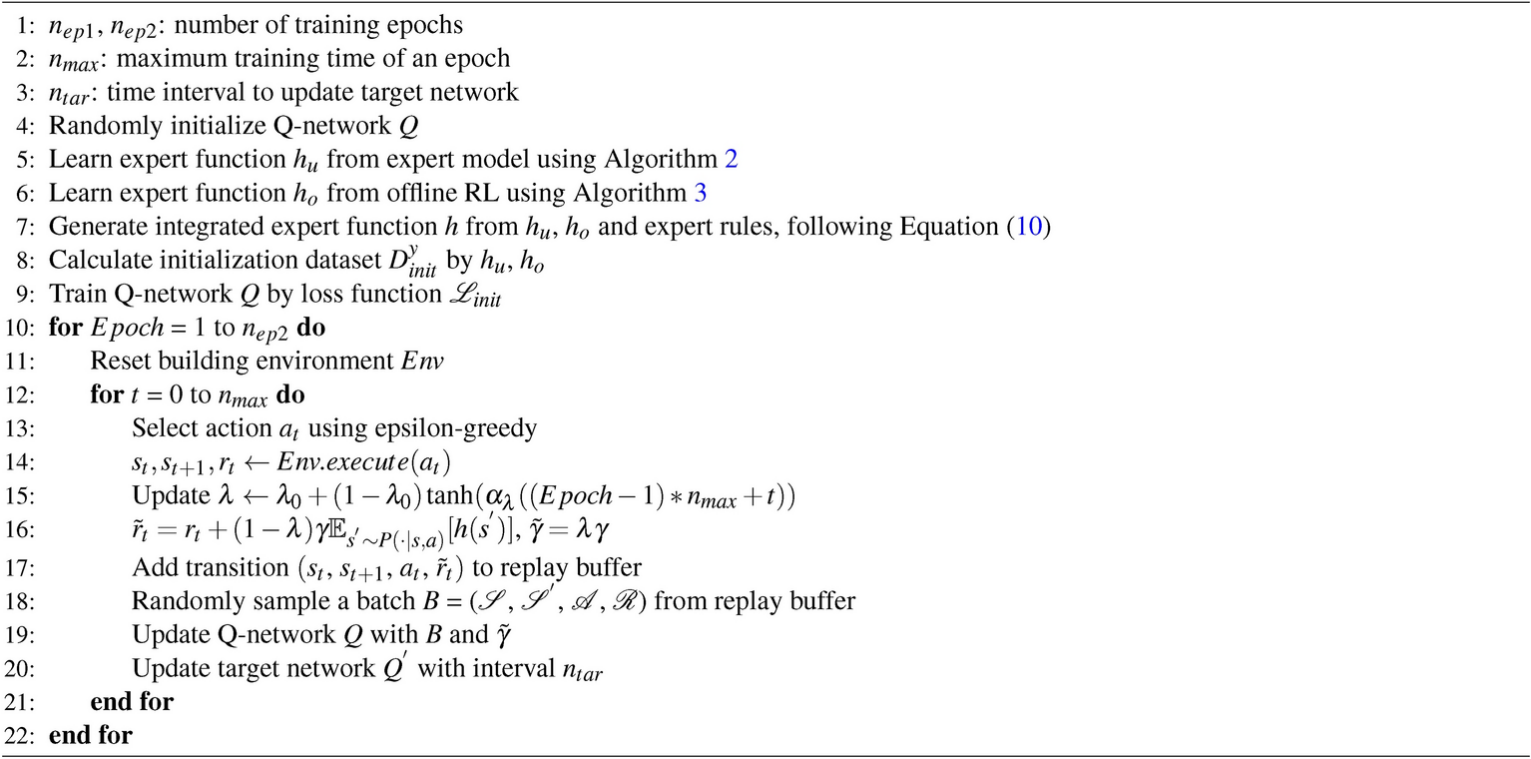
Our Online DRL Training Framework with Heterogeneous Expert Guidances

#### Underlying DRL algorithm

Similarly as in recent works^3,13,18^, we utilize double Deep Q-learning (DDQN)^44^ as the underlying DRL algorithm for our framework and also the baseline method for comparison in our experiments. We choose DDQN mainly for its convenience in leveraging the value function and the good performance it has shown for HVAC control in those recent works, but our expert-guidance approach can also be applied to improve the efficiency for other DRL algorithms.

We assume that the next state of the building HVAC system only relies on the current system state, and thus HVAC control can be treated as a Markov decision process (MDP). As stated in system model section, the state $s = (t, T^{in}_{t}, T^{env}_{t}, q^{sun}_{t}, P_{t}, T^{env}_{t+1}, T^{env}_{t+2},T^{env}_{t+3}, q^{sun}_{t+1}, q^{sun}_{t+2}, q^{sun}_{t+3})$. The discrete action space $\mathscr {A}$ contains the normalized air flow rate (0 to 1) with $m-1$ intervals. The reward is designed with consideration of indoor temperature violation and energy cost, as shown below:

1 $$\begin{aligned} r_t = \alpha \cdot \epsilon _t + \beta \cdot c_t, \end{aligned}$$

where $\epsilon _t$ represents the temperature violation for the current time step, $c_t$ is the energy cost for the current time step, and $\alpha , \beta$ are the scaling factors. More specifically, $\epsilon _t$ is defined as:

2 $$\begin{aligned} \epsilon _t = \max {(T^{in}_i - T_{upper}, 0)} + \max {(T_{lower} - T^{in}_i, 0)}, \end{aligned}$$

where $T_{upper}$is the upper bound of a given comfortable temperature range (which could be based on standards such as ASHRAE^45^or OSHA^46^) and $T_{lower}$ is the lower bound. Moreover:

3 $$\begin{aligned} c_t = p_t P_t, \end{aligned}$$

where $p_t$ is the energy price at time *t*, and $P_t$ is the power consumption during the current control interval at time *t*.

The goal of the DRL is to minimize total energy cost while maintaining indoor temperature within the comfortable temperature range. The loss function $\mathscr {L}_\mathscr{Q}$ for updating the Q-network is:

4 $$\begin{aligned} \mathscr {L}_Q = \mathbb {E}_{{(s_{t},a_{t},s^{'}_{t}) \sim D}}\left[ (r_t + \gamma \max _{{a_{t+1}}} Q^{'}(s_{t+1},a_{t+1}) - Q(s,a))^{2}\right] , \end{aligned}$$

where $s_t, s_{t+1} \in \mathscr {S}$, $a_t \in \mathscr {A}$, *Q* is the Q network and $Q^{'}$ is the target Q network. Then, the components introduced in the rest of this section will generate expert functions to provide prior guidance and policy initialization for this underlying DRL algorithm.

#### Learning expert function $h_u$ from expert model

An expert function $h_u$ can be learned through an expert model. In many cases, such expert model already exists in the form of an abstract physical model for the building thermal dynamics, e.g., an ARX or RC-networks model. While these abstract models are typically not accurate enough to enable good performance for DRL or model-based methods, they can be effectively leveraged to generate an expert function.

If an abstract physical model is not available, we can build a neural network as the expert model, with its parameters decided from a static historical dataset collected under existing control policy, as shown in Algorithm 2 (Line 5 in Algorithm 1) and described below.

**Algorithm 2:**
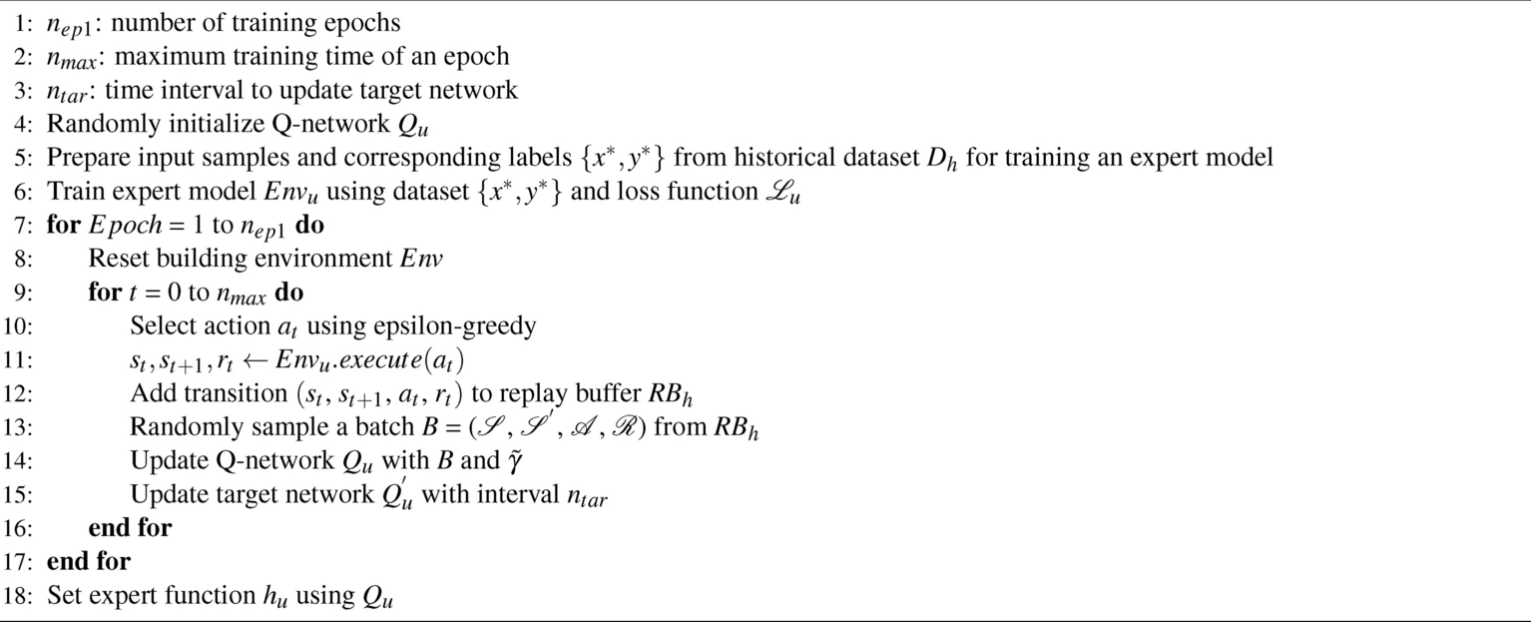
Learning Expert Function from Expert Model

We denote the historical dataset as $D_h$, with *n* data samples. For each data sample $(x, y) \in {D_h}$, let input $x = \{ t, T^{in}_{t}, T^{env}_{t}, q^{sun}_{t}, P_{t}, T^{env}_{t+1}, T^{env}_{t+2},$ $T^{env}_{t+3}, q^{sun}_{t+1}, q^{sun}_{t+2}, q^{sun}_{t+3}, a \}$ as defined in system model section and $a \in \mathscr {A}$, and let output label $y = \{T^{in}_{t+1}\}$. The neural network-based expert model consists of $m_{u}$fully-connected layers. All hidden layers are followed by a GELU activation function^47^, and are sequentially connected (the detailed layer setting will be specified later in Table 1 of experimental results section). Note that we choose this relatively simple MLP (multilayer perceptron) architecture because the network input is low-dimensional and the design already shows good performance in experiments. For large and complex multi-zone commercial buildings, RNNs (recurrent neural networks) or transformers could be promising architectures to explore in future work..

As different variables may not be in the same order of magnitude (e.g., *t* can be 1000 times larger than $T^{in}_t$), we normalize the input *x* and the output label *y*. The preprocessed input and output can be written as $x^* = \frac{x - x_l}{x_h - x_l}$, $y^* = \frac{y - y_l}{y_h - y_l}$, where $x_h$ and $x_l$ are the upper bound and lower bound of the variable *x*, and $y_h$ and $y_l$ are the upper and lower bound of the variable y. We then train the expert model with a mean square error loss function

5 $$\begin{aligned} \mathscr {L}_{u} = \parallel y^* - y^*_{pred} \parallel ^2, \end{aligned}$$

where $y^*_{pred}$ is the network prediction for the normalized *y*. When we apply this expert model after model training, we obtain the prediction of *y* by reversing the operation of previously-mentioned normalization step. It may not be necessary to predict the entire system state, e.g., the environment temperature $T^{out}_t$ and solar irradiance $q^{sun}_t$ may be obtained from weather forecast.

Once we have the expert model, either in the form of an abstract physical model or a neural network, the expert function $h_u$ can be viewed as a prior guess of the optimal value function in the building HVAC control task and can be learned via DRL. More specifically, we define an MDP problem $\hat{\mathscr {M}} = (\mathscr {S}, \mathscr {A}, \mathscr {P}_{u}, r, \gamma )$ where the definitions of state $\mathscr {S}$, action space $\mathscr {A}$ and reward function *r* are the same as defined at the beginning of underlying DRL algorithm section . $\mathscr {P}_{u}$ is from the expert model. We then apply DDQN on $\hat{\mathscr {M}}$ and obtain a trained Q-network *Q*. And the expert function $h_u$ can be set up as:

6 $$\begin{aligned} h_u(s) = \max _a{Q(s,a)}, \end{aligned}$$

where *s* is the state and *a* is the control action.

#### Learning expert function $h_o$ from offline RL

Another type of expert function $h_o$can be learned from the historical data via offline RL, as shown in Algorithm 3 (Line 6 in Algorithm 1). We leverage some of the techniques from conservative Q-learning (CQL)^39^ because of its effectiveness in reducing a large number of hyper-parameters.

**Algorithm 3:**
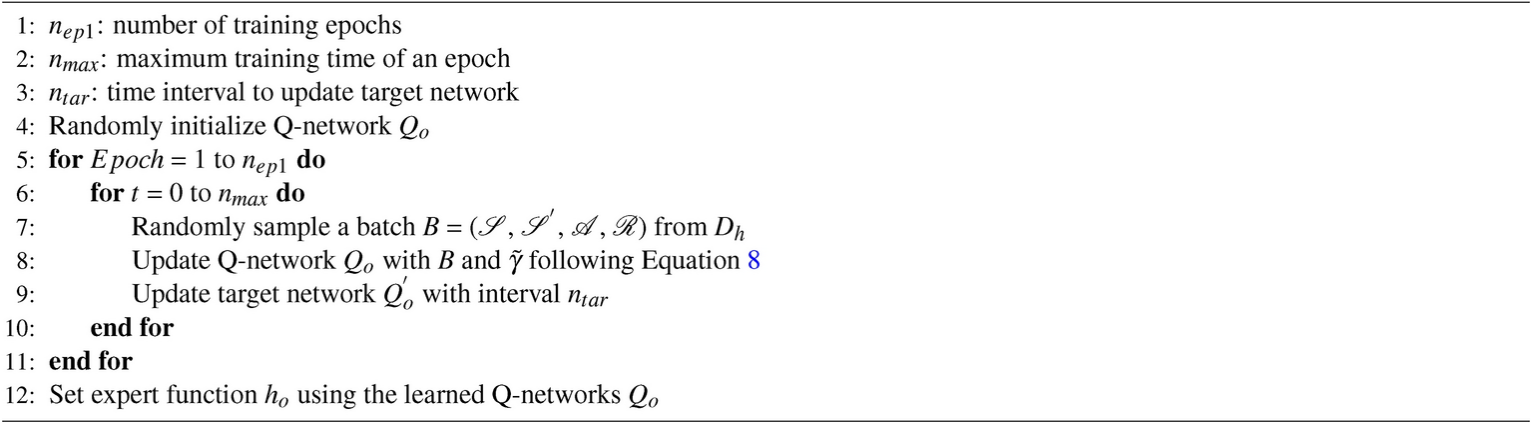
Learning Expert Function from Offline RL

First, we build an offline RL model based on DDQN, but with different Q-network updating rules as the DRL presented in the beginning of underlying DRL algorithm section. Compared with Equation (4), we add an extra regularization term:

7 $$\begin{aligned} \begin{aligned}&\mathscr {L}_{reg} = {\min _{Q} \mathbb {E}_{s_t \sim D}\left[ \log \sum _{a_t} \exp (Q(s_t, a_t))-\mathbb {E}_{a_t \sim \pi (a_t|s_t)}\left[ Q(s_t, a_t)\right] \right] }, \end{aligned} \end{aligned}$$

where $s_t \in \mathscr {S}$ and $a_t \in \mathscr {A}$. *Q* is the Q-network, and *D* is the dataset produced by the behaviour policy $\pi$. In the equation, the first part $\log \sum _{a_t} \exp (Q(s_t, a_t))$ describes a penalty term for minimizing the Q-value of the action produced by current policy on the states in the historical dataset. It helps learn a smaller and more conservative Q-value estimator. The second term $-\mathbb {E}_{a_t \sim \pi (a_t|s_t)}[Q(s_t, a_t)]$ counts average Q-value in the state-action pairs in the historical dataset and maximizes it to push the current learned policy closer to the behavior policy in the historical dataset.

Then the policy updating is changed as follows:

8 $$\begin{aligned} \begin{aligned} \mathscr {L}_{off} = \frac{1}{2}\mathbb {E}_{(s_t,a_t,s^{'}_t) \sim D}\left[ (r_t + \gamma \max _{a_{t+1}} Q^{'}(s_{t+1},a_{t+1}) - Q(s_t,a_t))^2\right] + \xi \mathscr {L}_{reg} , \end{aligned} \end{aligned}$$

where $s_t, s_{t+1} \in \mathscr {S}$, $\xi$ is a mixing coefficient, and $Q^{'}$ is the target Q-network. With enough training iterations, the offline RL agent can provide a good expert function $h_o$ following the same procedure as in Equation (6).

Note that we observe that not all offline RL algorithms can be a suitable choice for our framework. For example, approaches like TD3+BC^38^ may not always provide a good value estimation for the given states. We suspect that this may be due to two factors. One is related to the reward design, as the value function estimation in some offline RL algorithms is sensitive to the scale of the accumulated reward. The other is that because algorithms like TD3+BC only add regularization on the actor updating and do not set constraints on the Q function, which could enlarge the error in estimating the (Q-)value function when combined with possible numerical issues.

#### Generating integrated expert function *h* from $h_u$, $h_o$ and expert rules

The expert function $h_u$ learned from the expert model and the expert function $h_o$ learned via offline RL tend to perform differently because of the complexity of the system dynamic and the sufficiency of the data. Moreover, the accuracy of their Q-value estimation can vary at different states depending on the data distribution within the historical dataset. Thus, it is a natural thought to form an ensemble of the two. And the ensemble of multiple expert functions calculated in different ways can further reduce the overestimation of Q-values through a conservative way, which we will introduce in this section later.

To begin with, after having $h_u$ and $h_o$, we can combine them with *expert rules* to generate an integrated expert function *h*. The expert rules are often set by domain experts or building operators based on past experience and domain expertise. They do not provide an optimized control action for a given state, but instead offer suggestions that could be viewed as guidance or soft constraints – e.g., not turning on the cooling system when the indoor temperature is below the lower bound of the comfortable temperature range by certain threshold. Formally, we define that the expert rules $f_{rule}$ can generate an action candidate set $\textbf{U}_s$ for each state:

9 $$\begin{aligned} \textbf{U}_s = \{a | a \in f_{rule}(s), a \in \mathscr {A})\}. \end{aligned}$$

We can then generated an integrated expert function *h* based on $\textbf{U}_s$, $h_u$ and $h_o$ (Line 7 in Algorithm 1). Specifically, we apply a pessimistic ensemble strategy for selecting the value function estimation among different expert functions, and only choose corresponding actions from the expert rules’ action candidate set $\textbf{U}_s$. Thus, the integrated expert function *h* can be formulated as:

10 $$\begin{aligned} h(s) = \min _i(\max _{a \in \textbf{U}_s}{Q_i(s,a)}), \end{aligned}$$

where $Q_i$ is the Q-value estimation from expert functions *i*. Note that this is a *general formulation* that can unify multiple expert functions – e.g., we may have more than one abstract physical models that provide multiple $h_u$ expert functions.

#### Prior-guided learning

Once we have the integrated expert function *h*, we can use it to guide the underlying DRL with prior-guided learning. There are several algorithms that could guide online RL with a single prior policy, such as HuRL^48^and JSRL^49^. Our framework is flexible in choosing those and we select HuRL^48^ in our implementation. In the original HuRL, the Q-value estimation in the RL agent is guided by a simple heuristic function that is learned from the Monte-Carlo regression. In our work, we instead leverage the integrated expert function *h* from above. By dynamically changing a mixing coefficient $\lambda$ that controls the trade-off between the bias from the expert function *h* and the complexity of a reshaped MDP, we are able to accelerate the DRL training with a shortened MDP horizon. Specifically, given the state space $\mathscr {S}$, action space $\mathscr {A}$, reward function *r* that are mentioned at the beginning of underlying DRL algorithm section , as well as the transition dynamics of the building HVAC system $\mathscr {P}$ and a discount factor $\gamma$, we consider an MDP $\mathscr {M} = (\mathscr {S}, \mathscr {A}, \mathscr {P}, r, \gamma )$. We use the learned integrated expert function *h* as a prior guess for the optimal value function of $\mathscr {M}$. Thus our online DRL can be described as a reshaped MDP $\tilde{\mathscr {M}} = (\mathscr {S}, \mathscr {A}, \mathscr {P}, \tilde{r}, \tilde{\gamma })$, where $\lambda$ is a mixing coefficient,

11 $$\begin{aligned} \tilde{r} = r + (1-\lambda )\gamma \mathbb {E}_{s^{'} \sim \mathscr {P}(\cdot | s, a)}{[h(s^{'})]}, \end{aligned}$$

and

12 $$\begin{aligned} \tilde{\gamma } = \lambda \gamma , \end{aligned}$$

which is shown at Line 16 in Algorithm 1.

#### Policy initialization from expert function distillation

In the above section, we use the integrated expert function *h* to reshape the reward function and shorten the MDP horizon. In addition, we can also speed up the DRL training through better initialization, by leveraging the expert functions for determining the initial policy (Lines 8 and 9 in Algorithm 1).

Specifically, we initialize the deep Q-network through knowledge distillation^50^ on the expert functions. The first step is to extract the knowledge from multiple expert functions ($h_u$ and $h_o$ in our case) to a dataset $D_{init}$. We set the input dataset as $D^x_{init}$ and the corresponding label set as $D^y_{init}$. In setting $D^x_{init}$, we utilize all the unlabeled historical data, which only contain the system state. And the corresponding labels are calculated in a way that is similar to the strategy introduced earlier for integrating expert functions. That is, suppose we have $n_h$ expert functions, then

13 $$\begin{aligned} D^y_{init}&= \{y | y = (q_1, q_2, \cdots , q_m)\}, \end{aligned}$$

14 $$\begin{aligned} q_j&= \min _i({Q_i(s,f_j)}), \end{aligned}$$

where $s \in D^x_{init}, j \in [1 \cdots m], i \in [1 \cdots n_h]$. As the expert functions we utilize are not as accurate as of the optimal (Q-) value function, we further add two mixing coefficients $\lambda ^\alpha _{init}$, $\lambda ^\beta _{init}$ for balancing the relative size of the Q value from different actions. So the new definition of $D^y_{init}$ is

15 $$\begin{aligned} \begin{aligned} D^y_{init} = \left\{ y | y = \left( \frac{q_1+(\lambda ^\alpha _{init} - 1)\mu _q}{\lambda ^\alpha _{init}\lambda ^\beta _{init}}, \frac{q_2+(\lambda ^\alpha _{init} - 1)\mu _q}{\lambda ^\alpha _{init}\lambda ^\beta _{init}}, \cdots , \frac{q_m+(\lambda ^\alpha _{init} - 1)\mu _q}{\lambda ^\alpha _{init}\lambda ^\beta _{init}}\right) \right\} , \quad \mu _q = \frac{\sum _{j=1}^m{q_j}}{m}, \end{aligned} \end{aligned}$$

where the definition of $q_j (j \in [1 \cdots m])$ remains the same. Then the next step is to train the deep Q-network of our DRL agent by using the obtained dataset $D_{init}$. As we consider a regression task, we apply the mean square error as the loss function

16 $$\begin{aligned} \mathscr {L}_{init} = {\parallel y - y_{pred} \parallel }^2, y \in D^y_{init}, \end{aligned}$$

where $y_{pred}$ is the deep Q-network prediction. We obtain the network weight initialization by training for $n_{init}$ epochs. Moreover, with such policy initialization, we can use a smaller learning rate to tune the deep Q-network in the later DRL stages.

### Runtime shielding framework

**Fig. 2:**
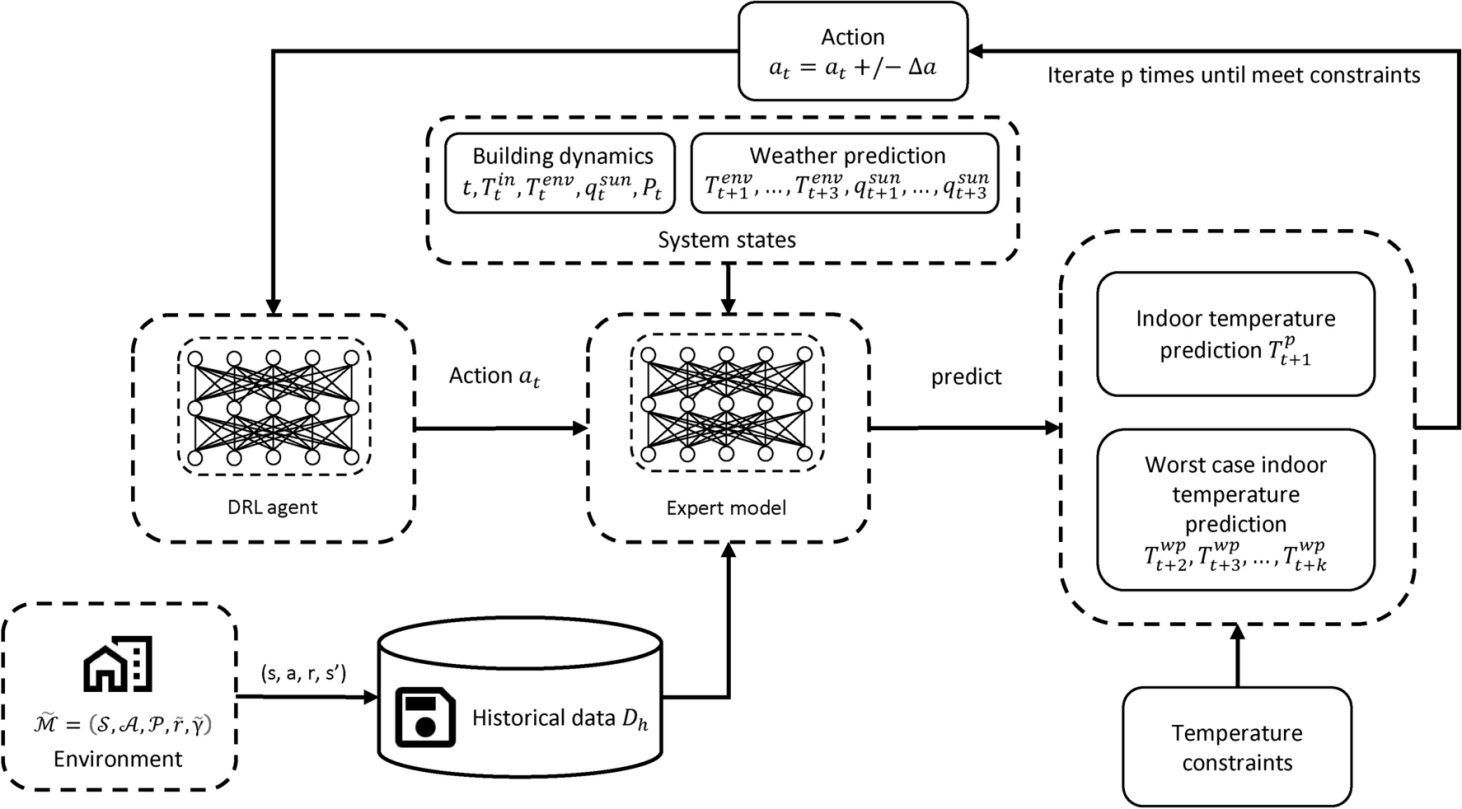
Overview of our runtime shielding framework for reducing temperature violation rate. The framework includes two major components: 1) the learned DRL agent (by our online DRL training framework as introduced earlier) that produces the control action based on the current system state, and 2) an expert model for predicting indoor temperature in future steps, based on a neural network with its parameters determined from historical data. More specifically, the expert model takes the current system states and proposed action from the DRL agent as input, and predicts the indoor temperature for the next step and the worse-case indoor temperature for the next few steps. Based on such predictions, the control action may be adjusted iteratively to meet the temperature constraints.

As shown in the previous sections, while the training of our DRL agent considers both the temperature violation and the energy cost in the reward function design, there is no explicit enforcing of the constraints on comfortable temperature range. Similarly for many other learning-based (and model-based) controllers, there is no explicit enforcing of the temperature constraints on the control actions, which may lead to constant temperature violations. Thus, in this work, we propose a novel runtime shielding framework to help HVAC controllers meet temperature constraints. The framework does not affect the controller training process and is agnostic to the controller design.

Figure 2 shows the overview of our runtime shielding framework, which integrates the HVAC controller – in this case, the DRL-based controller trained by our proposed online framework under heterogeneous expert guidances – with an expert model that predicts future indoor temperature based on the system states and the control input. The expert model is trained from the historical data collected from the building environment, similarly as the one used in our online training framework but with different goal and output. More specifically, during runtime, the learned DRL agent proposes a control action $a_t$ for the current time *t*. The expert model for temperature prediction takes the proposed control action $a_t$ from the DRL agent and the system states $s_t$ as input, and outputs the indoor temperature prediction that includes not only the temperature prediction for the next time step $T_{t+1}^p$ but also the *worst-case* temperature prediction from time $t+2$ to $t+k$, named as $T_{t+2}^{wp},\dots , T_{t+k}^{wp}$.

In particular, at time $t+i$ ($2 \le i \le k$), the expert model predicts the worst cases regarding both the temperature upper bound and the temperature lower bound as follows: (1) Based on the predicted temperature at time $t+i-1$ and the worst-case control action for temperature lower bound at time $t+i-1$, which is $a^{wp,l}_{t+i-1} = 0$, the expert model will predict the indoor temperature at time $t+i$, named $T^{wp,l}_{t+i}$. This is a worst-case prediction of the temperature lower bound, i.e., $a_t = \max (a_t - \Delta a, 0),$ if $T^{wp,l}_{t+i} < T_{lower}$. (2) On the other hand, based on the predicted temperature at time $t+i-1$ and the worst-case control action for temperature upper bound at time $t+i-1$, which is $a^{wp,u}_{t+i-1} = m-1$, the expert model will predict the indoor temperature at time $t+i$, named $T^{wp,u}_{t+i}$. This is the worst-case prediction of the temperature upper bound, i.e., $a_t = \min (a_t + \Delta a, m-1),$ if $T^{wp,u}_{t+i}> T_{upper}$. Then, the comfortable temperature range will serve as the constraints against which these temperature predictions are checked. If the predictions are out of the range, the current proposed control action $a_t$ will be iteratively adjusted until the temperature constraints are met or the iteration number reaches *p*. Moreover, if such change for the proposed control action based on the prediction of time $t+i$ makes the previous temperature predictions (i.e., $T^p_{t+1}$, $T^{wp}_{t+2}, \dots , T^{wp}_{t+i-1}$) violate the temperature constraints, we will discard the result, stop next worst-case predictions, and use the results from time $t+i-1$.

## Experimental results

### Experiment settings

**Table 1:** Hyper-parameters used in our experiments.

Parameter	Value	Parameter	Value
Expert- model	[$len(s \in \mathscr {S})$, 256,256,256, 256,256,256,2]	Deep Q- network	[$len(s \in \mathscr {S})$, 256, 256, 256, 256, 51]
*m*	51	$\Delta t$	15 mins
$\gamma$	0.99	$\alpha$	1.0
$T_{lower}$ (occupied)	$22^{\circ }\hbox {C}$	$T_{upper}$ (occupied)	$26^{\circ }\hbox {C}$
$T_{lower}$ (unoccupied)	$12^{\circ }\hbox {C}$	$T_{upper}$ (unoccupied)	$30^{\circ }\hbox {C}$
$\beta$	100.0	$m_{u}$	7
$\xi$	1.0	n	5760

**Fig. 3:**
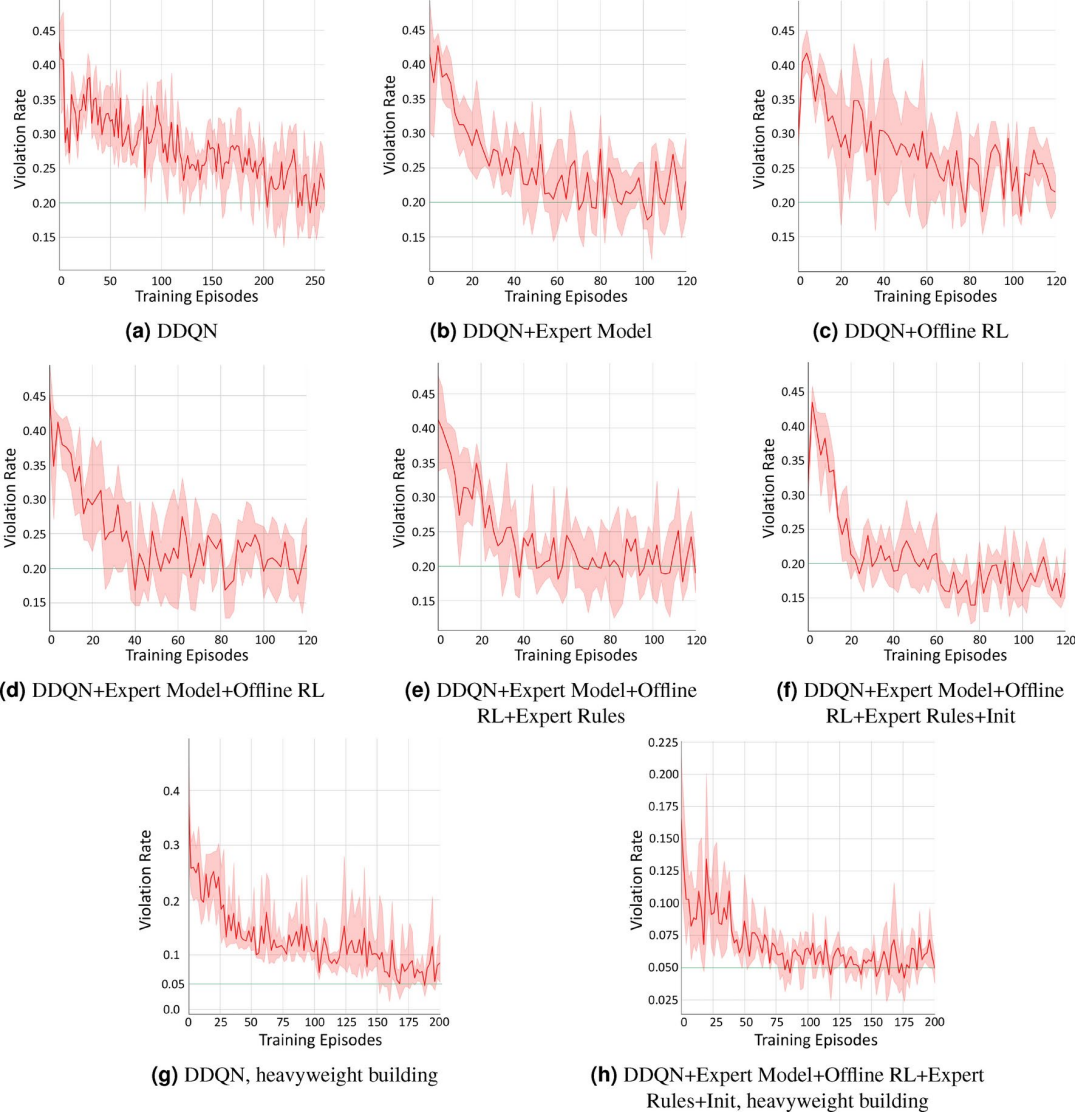
Figure 3a to Figure 3f show the comparison between our online DRL training framework (in different settings with various techniques included) and the standard DDQN method on the lightweight building. The weather data is from Riverside, CA, USA. The x-axis shows the training episodes. The y-axis shows the temperature violation rate. Figure 3a shows the training process under the standard DDQN method. About 212 episodes are needed to reach a violation rate of 0.2. Figure 3b, Figure 3c, Figure 3d, and Figure 3e show the results when we gradually add an expert model that generates expert function $h_u$, offline RL that generates expert function $h_o$, an expert rule, and policy initialization based on expert functions, respectively. And we can observe the improvement on the required episodes step by step. Figure 3f shows the training process when we apply all of our techniques. In this case, only 24 episodes are needed to reach the violation rate of 0.2, an 8.8*X* improvement over standard DDQN. Then, Figure 3g and Figure 3h show the comparison between our approach with all techniques included (right) and the standard DDQN baseline (left) on the heavyweight building with larger thermal capacity under the weather data from Chicago, IL, USA. The green bar in each sub-figure shows the target temperature violation rate, depending on the building type. The number of training episodes is chosen to ensure that the violation rate has reached around or below the target level and it has relatively plateaued.

We conduct our experiments on a Ubuntu 20.04 OS server equipped with NVIDIA RTX A5000 GPU cards. Docker^51^is utilized for the environment configuration, with Python 3.7.9 and learning framework Pytorch 1.9.0. All neural networks are optimized through the Adam optimizer^52^.

We use the building simulation tool in paper^18^to simulate the behavior of single-zone commercial buildings, with an OpenAI-Gym^53^interface. We model two buildings as defined in the Building Energy Simulation Test validation suite^54^: one is with a lightweight construction (known as Case600FF) and the other is with a heavyweight construction (known as case900FF). Both buildings have the same model settings except that the wall and floor construction have either light or heavy materials. The floor dimensions are 6*m*-by-8*m* and the floor-to-ceiling height is 2.7*m*. There are four exterior walls facing the cardinal directions and a flat roof. The walls facing east-west have the short dimension. The south wall contains two windows, each 3*m* wide and 2*m*tall. The use of the building is assumed to be a two-person office with a light load density. The lightweight building is assumed to be located at Riverside, California, USA, and the heavyweight building is assumed to be located at Chicago, Illinois, USA. The weather data for different locations are obtained from the Typical Meteorological Year 3 database^55^. In addition, the various parameters and hyper-parameters mentioned in the previous sections are listed in Table 1.

### Evaluation of our online DRL training framework and comparison with standard DDQN

We first apply our proposed online RL framework with heterogeneous expert guidances to building HVAC control and demonstrate its effectiveness in accelerating the DRL training, in particular the standard DDQN algorithm. We repeat each experiment 4 times and show the average results.

#### Comparison with standard DDQN on training efficiency

Figure 3demonstrates the temperature violation rate of the trained controller under different approaches for the lightweight building with weather data from Riverside. Temperature violation rate is one of the main objectives for DRL. It is defined as the percentage of the time the indoor temperature is outside of the comfortable temperature zone, similarly as used in works^3,6,13,18^.

Figure 3a shows the training process of the standard DDQN, and the model needs **about 212 episodes to reach a violation rate at around 20%**for this building from^18^ ($20\%$ may seem high, but it is due to the limitation of this particular building and its cooling-only HVAC system; more explanation on this later with Figure 5). Figure 3b shows the training process when we add a neural network-based expert model that generates the expert function $h_u$. About 68 episodes are needed to reach the same violation rate. Figure 3c shows the training process when we add offline RL that generates the expert function $h_o$, and about 78 episodes are needed to reach the violation rate of $20\%$. Figure 3d shows the results when we apply both expert functions $h_u$ and $h_o$, but without the expert rules. We can see that about 40 episodes are needed. Figure 3e shows the results when we integrate the two expert functions $h_u$ and $h_o$, as well as an expert rule *f* using the method introduced in Equation 10 . *f* is defined as follows: when the indoor temperature is below $22^{\circ }\hbox {C}$, the control action is suggested to be set within the set of $\{f_0, f_1, f_2, f_3\}$; if the indoor temperature is above $27^{\circ }\hbox {C}$, the control action is suggested to be set within the set of $\{f_{m-3}, f_{m-2}, f_{m-1}, f_m\}$. We can see that the number of episodes needed is about 36. Finally, Figure 3f shows the training process when we apply all of our proposed techniques, including integrating the expert functions from expert model and offline RL as well as the expert rules, using the integrated expert function to guide DRL training, and conducting policy initialization with the expert functions. We can see that **now only 24 episodes are needed to reach the same violation rate as the standard DDQN, an 8.8X reduction in training time**. Table 2 summarizes the above number of episodes required to reach the violation rate of 0.2 for the standard DDQN baseline and our approach with various techniques included.

**Table 2:** Number of episodes required on the lightweight building to first reach the violation rate of 0.2 for the standard DDQN baseline and our online DRL training framework with various techniques included, corresponding to the results in Figure 3a to Figure 3f (the last line being our approach with all techniques in Algorithm 1).

Method	Number of Episodes
DDQN	212
DDQN+Expert Model	68
DDQN+Offline RL	78
DDQN+Expert Model+Offline RL	40
DDQN+Expert Model+Offline RL	36
+Expert Rules	
DDQN+Expert Model+Offline RL	24
+Expert Rules+Init	

For further evaluation, we also conduct experiments on the heavyweight building with weather data from Chicago. In this set of experiments, the major change of the parameters is that the scaling factor $\beta$ is set to 1.0 in Equation (1). This is because that the average energy consumption of this HVAC system is much higher than that of the previous building, and we need to re-balance the energy cost and the temperature violation in the reward design. Figure 3g and Figure 3h shows the comparison between our approach and the standard DDQN. And the experiments show that the number of episodes needed to first reach a violation rate of $5\%$ is reduced from around 160, as shown in Figure 3g, to around 80 in Figure 3h. The improvement, while still significant, is much less than the lightweight building. We suspect that this may be due to the quality of the historical data and plan to investigate it further in future work.

#### Energy cost and other details

Besides temperature violation rate and the number of episodes for reaching the goal of violation rate below 0.2 (i.e., training efficiency), we also assess the energy cost of the learned controllers during our experiments. We observed that different methods, including the standard DDQN baseline and our approach with various techniques included, achieve very similar energy cost for the learned controllers – in fact within $1\%$ for both the lightweight building and the heavyweight building we tested.

**Fig. 4:**
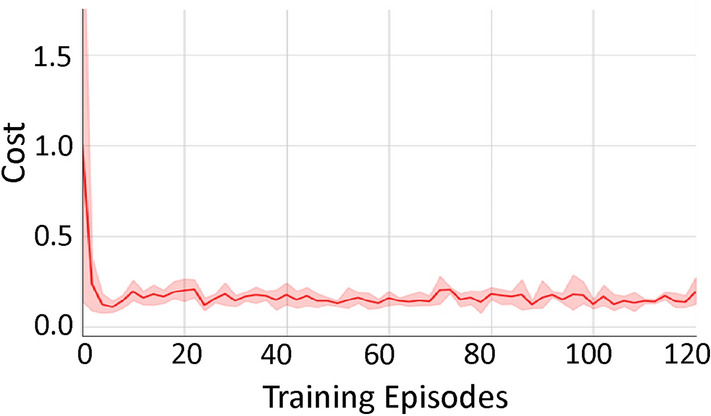
Normalized energy cost during training for our approach with all techniques included for the lightweight building with weather data from Riverside.

Figure 4 shows the normalized energy cost of our approach with all techniques included for the lightweight building with weather data from Riverside. We can observe that the energy cost quickly decreases to a lower value within 5 to 10 epochs and slightly fluctuates in the later training epochs.

**Fig. 5:**
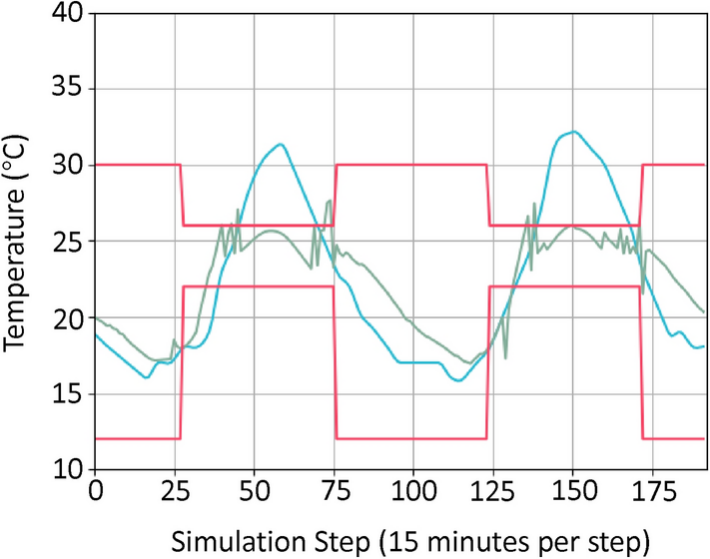
An illustration of the lightweight building temperature over 2 days under the controller learned from our approach with all techniques included. The red lines bound the comfortable temperature range. The blue line is the outdoor temperature in Riverside, CA. The green line is the indoor temperature under the learned controller.

Figure 5 illustrates the building temperature over 2 days, under the controller learned with our approach with all techniques included, for the lightweight building with weather data from Riverside. We can see that the temperature violation rate is around $20\%$. It is relatively high because some violations are very hard to avoid for this particular building. Specifically, the HVAC system is set to only work during the occupied hours (from 7am to 7pm) and the comfortable temperature range is much more strict during that time ($22^{\circ }\hbox {C}$ to $26^{\circ }\hbox {C}$) compared to during the unoccupied time ($12^{\circ }\hbox {C}$ to $30^{\circ }\hbox {C}$)^18^. This makes it almost impossible to meet the comfortable temperature range early in the morning since the HVAC system only provides cooling. We can see that after the early morning hours, the temperature is controlled well within the comfortable range by our controller.

### Ablation studies

#### Impact of the historical data quantity

We are interested in knowing how the quantity of the historical data may affect the performance of our approach. We conduct a series of experiments that have the quantity of the historical data chosen from $\{5760, 2880, 1440, 720\}$ (i.e., from 2 months of data to 7.5 days of data). The results are shown in Table 3. We can observe that the training becomes faster as the quantity of the historical data becomes larger, as what we would expect.

**Table 3:** The number of epochs needed by our approach (with all techniques included) for reaching the violation rate of $20\%$ for the lightweight building, under different quantity of the historical data.

#Samples	720	1440	2880	5760
#Episodes	116	78	62	24

#### Impact of the control quality of historical data

We also study the performance of our approach under different levels of control quality of the historical data. Previously we directly use the historical data collected from an existing controller on the target building. To study different control quality of such historical data, we choose to take random actions with a probability of *p* – intuitively, higher *p* values implies more random control and hence worse quality. Table 4 shows the results. Our approach performs better with a smaller *p*, i.e., when our approach learns from historical data based on more reasonable control actions.

**Table 4:** The number of epochs needed by our approach (with all techniques included) for reaching the violation rate of $20\%$ for the lightweight building, under different control quality of the historical data.

*p*	1.0	0.8	0.4	0.2	0.0
#Episodes	110	104	88	60	24

#### The usage of abstract physical model

**Fig. 6:**
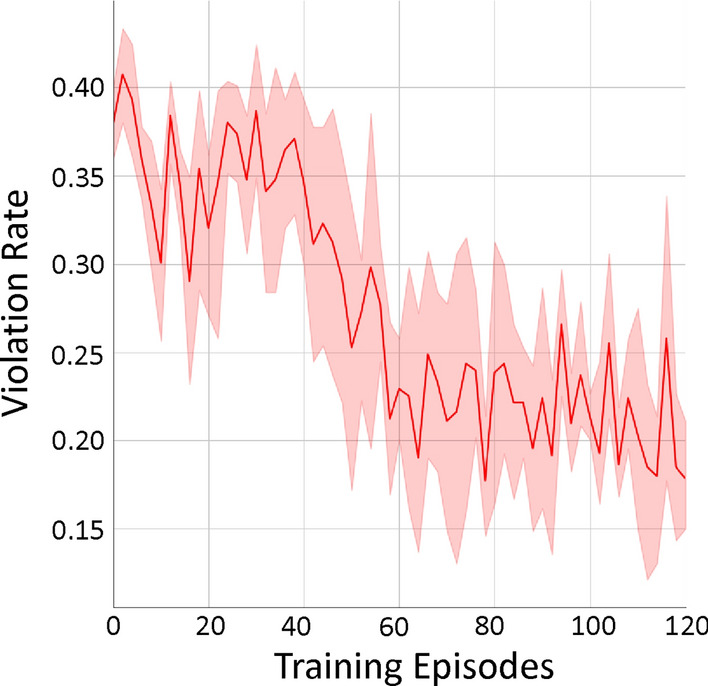
Training result for the lightweight building when the expert model in our approach (with all techniques included) is constructed from an abstract physical model.

In addition, we also try to utilize an abstract physical model, i.e., the ARX model from^6^, as the expert model to generate $h_u$, instead of learning a neural network. The training process is shown in Figure 6. About 64 episodes are needed to reach the same violation rate, more than the case where the expert model is a neural network learned from historical data. We think that this is due to the simplicity of the ARX model, and plan to investigate the performance of other abstract physical models in future. Nevertheless, it still provides considerable improvement over the standard DDQN.

### Evaluation of our runtime shielding framework

For evaluating our proposed runtime shielding framework, in particular its capability in further reducing the temperature violation rate for our learned DDQN-based DRL controller, we conduct experiments on the heavyweight building. The expert model predicts the indoor temperature for the next step and the worst-case indoor temperature for another step ahead. We consider two DDQN agents – agent 1 is coarsely trained and agent 2 is the final model after additional training (the same one shown in Figure 3h).

From the results shown in Table 5, we can observe that while the temperature violation rate of agent 1 is initially quite high as it is coarsely trained, our runtime shielding framework can significantly decrease the violation rate by more than 3*X* (from $26.56\%$ to $7.81\%$), with only slight increase in energy cost. For agent 2, which has a much lower temperature violation rate than agent 1 given the additional training, our runtime shielding framework can still reduce the violation rate substantially, from $4.17\%$ to $3.65\%$, with slight reduction on energy cost as well (Note that further reduction on temperature violation rate for this particular system is very challenging, due to the cooling-only nature of its HVAC system. As stated before, there is always a short period in the early morning when the indoor temperature is below the desired lower bound and the HVAC system has just started, as shown in Figure 5. Thus, without adding heating, there will be at least 2%−3% temperature violation rate for the system.). Overall, these results for agent 1 and agent 2 demonstrate the **effectiveness of our shielding framework in reducing temperature violation rate while keeping similar energy cost**, for controllers that have been trained to different degrees and have varying qualities.

**Table 5:** Comparison between two DDQN agents (trained by our online DRL training framework to different degrees) and when they are incorporated into our runtime shielding framework, in both temperature violation rate and energy cost.

Method	Temperature Violation Rate (%)	Energy Cost
DDQN agent 1	26.56	1.60
DDQN agent 1 + Runtime Shielding	7.81	1.68
DDQN agent 2	4.17	2.07
DDQN agent 2 + Runtime Shielding	3.65	1.94

### Experiments in other domains

**Fig. 7:**
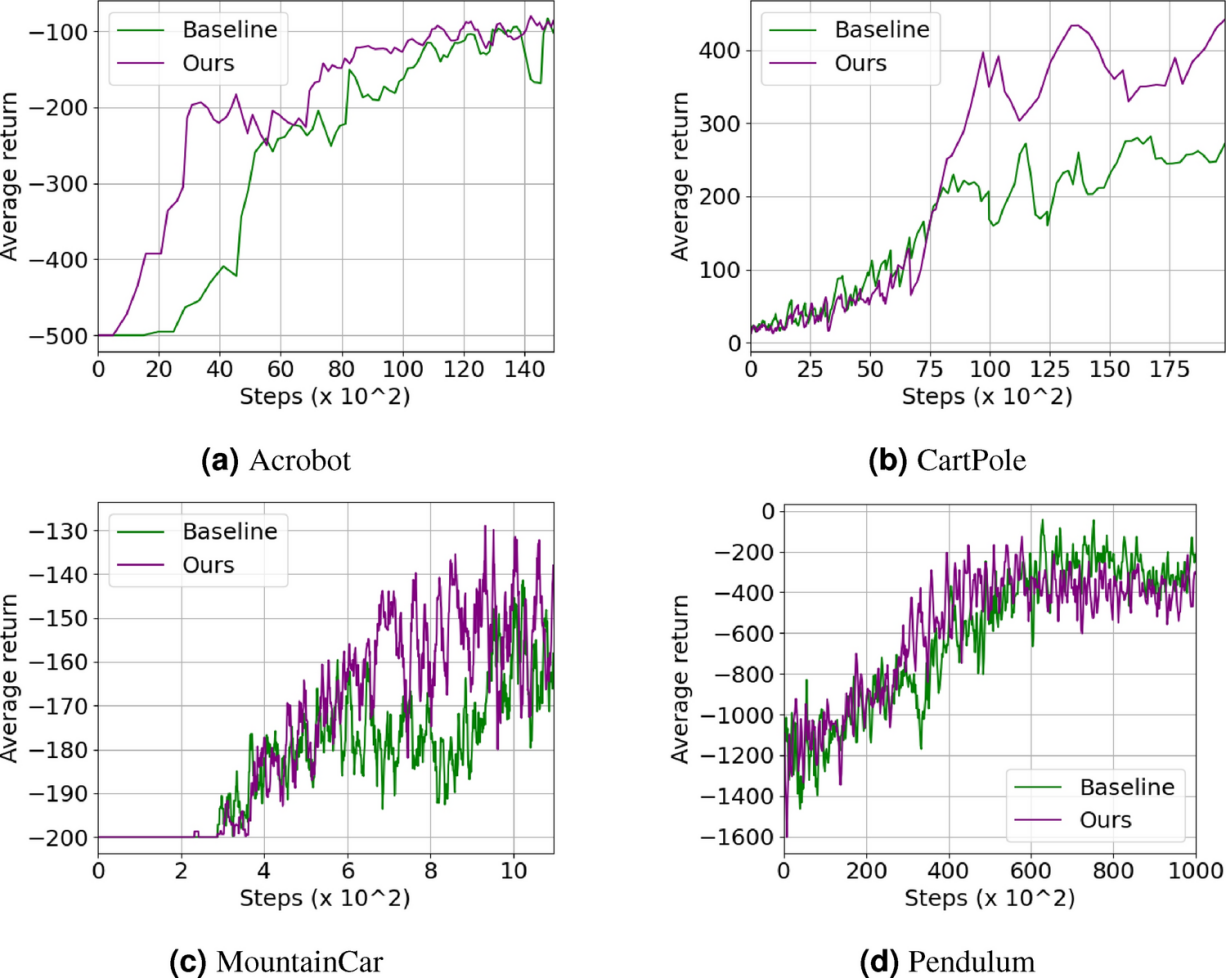
Results on examples from the Gym environments.

We believe that our approach of leveraging existing domain expertise in DRL training may be extended to other domains of cyber-physical systems. Thus, we conduct initial exploration outside of the building domain, on a few examples from the Gym^56^ environment, to assess our approach’s general applicability. Figure 7 shows the comparison between our approach and the standard DDQN baseline. We observe that our approach is able to significantly improve the learning efficiency and/or performance on some examples (particularly CartPole) but not others (Pendulum in particular). We think that in these cases the final results can be significantly affected by the quality of the generated expert functions. First, the offline data collected can affect the quality of both the expert model and the offline RL component. Since the task environment is different, the difficulty of constructing a good expert model from the historical data is also an important factor. Compared with the accuracy of predicted system states, the accuracy of the terminate condition in each step can also have a significant impact in some tasks. However, the terminate condition is always fixed in building HVAC control (run for certain steps) and we can even utilize the prior knowledge from the building domain combined with the offline data to help construct the expert model. These factors make the building HVAC control an ideal application for our approach.

## Conclusions

In this paper, we present a systematic, unified framework to accelerate online RL for building HVAC control with heterogeneous expert guidances, including abstract physical models, historical data, and expert rules. These guidances are unified through the learning of expert functions, which are then used to accelerate DRL with prior-guided learning and policy initialization. Moreover, we propose a runtime shielding framework for further reducing the temperature violation. A series of experiments demonstrate that our approach can significantly reduce the training time over previous DRL methods while maintaining the indoor temperature within the comfortable temperature range. We believe that our approach not only addresses a critical challenge in applying DRL to building domain, but also has the potential in other domains where existing expertise could be leveraged in improving learning efficiency and performance. We plan to investigate this further in future work.

## Data Availability

All the data we used are generated from the building simulation platform^18^, which is publicly available in https://github.com/YangyangFu/mpc-drl-tl.
